# Effect of Enzyme Pre-treatments on Bioactive Compounds in Extracted Tiger Nut Oil and Sugars in Residual Meals

**DOI:** 10.1007/s11746-016-2883-9

**Published:** 2016-08-26

**Authors:** Onyinye Ezeh, Keshavan Niranjan, Michael H. Gordon

**Affiliations:** Department of Food and Nutritional Sciences, University of Reading, Whiteknights, PO Box 226, Reading, RG6 6AP UK

**Keywords:** Tiger nut oil, Enzymes, High pressure processing, Sugars, Pressing

## Abstract

Tiger nut oil is a novel oil that requires more research data on its characteristics. In this study, the oil was extracted using both enzyme-aided pressing (EAP) and aqueous enzymatic extraction (AEE) methods. Using enzymes as a pre-treatment prior to mechanical pressing increased the concentration of some phenolic acids and tocopherols present in extracted oils compared to controls. High pressure processing as a pre-treatment before aqueous enzymatic extraction also enhanced tocopherols and total polyphenolic content in oils. The percentage free fatty acid and peroxide values indicated that under the initial extraction parameters, the oils were stable and they all met the standards for virgin olive oil set by the International Olive Oil Council. Residual meals from both extraction processes contained low protein contents ranging from 2.4 to 4.6 %. Additionally, EAP and AEE meals contained low DP (degree of polymerisation) sugars that appeared as 1-kestose (DP3) and nystose (DP4). EAP had the highest total DP3 and DP4 sugar content of 82.5 mg/g. These sugars would need further assessment to verify their identity and determine their suitability as a potential food.

## Introduction

Quite often, claims for a number of beneficial qualities to different aspects of life ranging from human health to hair and skin care are made for some plant oils [1, 2]. These benefits are commonly attributed to the composition and chemical constituents of the oils. An example is the high lauric acid content of coconut oil which allows it to easily penetrate into hair shafts, and together with coconut oil’s affinity for protein, offers protection from hair protein loss [2]. Applications of this oil are not limited to hair care, but also used in cooking, baking and frying as well as skin preparations. Similarly, olive oil is as versatile, because of its fatty acid composition, and content of bioactive compounds such as polyphenols and Vitamin E.

With the numerous possible sources of plant oils, it is important to identify new sources in order to diversify the range available to consumers and encourage local production in areas where the plants are grown. Furthermore, development of new sources allows sustainable growth, manufacture and use of the oils. Tiger nut oil, although not entirely new, remains unknown in major parts of the world and underutilised even in regions where it is cultivated. It has already been described as being similar to olive oil in literature [3]. Tiger nut is a tuber of the *Cyperus esculentus* plant and the cultivated variety is generally identified as Chufa. It is widely distributed across the world and popular in countries such as Spain, Egypt, and Nigeria to name a few. In these countries, it is consumed as a snack and used to make cold beverages. Its oil is commonly used as a cooking ingredient, and in skin care [3]. Commercially, the oil exists as a cold-pressed oil [3]. Research on the extraction and characterisation of the oil is limited and only a few studies exist [3].

Oil extraction is typically conducted using solvent extraction with *n*-hexane as the commonly used solvent. However, with the concerns regarding solvent extraction such as the use of flammable solvents, and toxicity of residual meals, other methods of oil extraction are currently being explored especially environmental friendly alternatives [4], some of which includes aqueous extraction processes. The drawback of these methods is their low oil yields and pre-treatments are often employed to improve the process. The use of enzymes is becoming a popular tool to increase oil yields as they act to degrade cellular components of oil bearing materials and thus aid oil extraction [4]. Enzymes that have been used include proteases, cellulases, and hemicellulases, depending on the oil bearing material [4].

High pressure processing is commonly regarded as a medium for food preservation as the high pressures exerted on food systems can destroy spoilage inducing microorganisms. It has however been used as a pre-treatment tool in the enzymatic aqueous extraction of oil from soybean [5]. Studies into the effect of this technology in the extraction of tiger nut oil had been conducted and evaluated in previous research [4]. In studies conducted in our laboratory, it was found that it increased the oil yield during aqueous enzymatic tiger nut oil extraction.

Amongst the several factors that affect quality of oils during the extraction process, the processing conditions employed is one of them. It is rather important that key functional components of the oil are retained when the processing conditions are altered including bioactive compounds such as tocopherols and polyphenols. Tocopherols have been described as the most important natural group of antioxidants found in vegetable oils [6]; not surprising as it participates in different pathways as an antioxidant including acting synergistically with ascorbic acid all with the end result of preventing lipid peroxidation. Another important aspect of oil extraction processing is the use of the residual meals. Commonly, they are popular as animal feed because of the high protein content that most oilseeds possess. To properly utilise them, an assessment of their components needs to be done. In this study, tiger nut oil was extracted using both enzyme aided pressing (EAP) and aqueous enzymatic extraction (AEE) methods. The use of enzymes had been shown in a study conducted in our laboratories to improve oil yields [4]. The extracted oils were evaluated for their chemical composition and quality. Bioactive compounds present in the oils were assessed. Residual meals from the processes were also evaluated for their soluble sugars composition.

## Materials and Methods

### Samples

Oils were extracted using EAP and AEE methods. To prepare the samples, whole tiger nut tubers from Spain were soaked in distilled water for 6 h, ground using a coffee mill and sieved to a particle size of ≤0.425 mm using ASTM standard sieves. The ground samples were then used for oil extraction as described below.

### Enzymes

Alcalase (from *Bacillus licheniformis*), α-amylase (*B. licheniformis*), Viscozyme^®^ L (*Aspergillus*) (hemi cellulolytic enzyme mixture from *Aspergillus*) and Celluclast ^®^ 1.5 L (*Trichoderma reesei*) were purchased from Sigma-Aldrich, UK. Their activities and optimum conditions have been given in Table 1.

**Table 1:** Conditions for enzyme aided and aqueous enzymatic extraction of tiger nut oil

	Enzyme aided pressing	Aqueous enzymatic extraction
Enzymes	Amylase, alcalase, viscozyme	Amylase, alcalase, celluclast
Enzyme concentration (%, w/w)	1.0	0.5
Agitation speed (linear strokes/min)	120	120
Solid/liquid ratio	1:1.7	1:4

### Enzyme Aided Pressing

The extraction process illustrated in Ezeh, Gordon and Niranjan [4] was as follows: a combination of Alcalase, α-amylase and Viscozyme was used in the ratio 1:1:1. The operating conditions used are shown in Tables 1 and 2. Enzymes were added to distilled water, 30 g of ground tiger nut sample, and pH was adjusted to 8 using 0.5 M NaOH. Incubation was carried out for 6 h at 40 °C in a shaking water bath (Grant OLS, Cambridge, UK). After incubation, the mixture was dried in a vacuum oven till the moisture content was between 6.5 and 8 %. Temperature in the oven was 55 °C while the maximum pressure reached was 700 mmHg. Following drying, oil was extracted by mechanical pressing. Controls consisted of samples extracted with the same method without the use of enzymes. Starch present in the nuts led to the use of α-amylase and protease, while Viscozyme was added to aid in softening cell wall structure for the pressing process.

**Table 2:** Enzymes, activity and hydrolysis condition

Enzyme	Main activity	Recommended pH range	Recommended temperature range (^o^C)	pH and temperature used.
α-Amylase (*Bacillus licheniformis*)	Endoamylase	7–9	40–60	8, 40 °C
Celluclast^®^ 1.5 L (*Trichoderma reesei*)	Cellulase	4.5–6.0	50–60	5, 50 °C
Viscozyme^®^ L (*Aspergillus*)	Mixture of carbohydrases including xylanase, arabanase, cellulase, b-glucanase, and hemicellulase	3.3–5.5	40–50	4, 40 °C
Alcalase (*Bacillus licheniformis*)	Endo-protease	7.5–9.0	45–60	8, 50 °C

### Aqueous Enzymatic Extraction

Ground nuts were weighed into 200-ml Duran bottles followed by addition of enzymes and distilled water (Table 1). The bottle was shaken to mix thoroughly before pH was adjusted to pH 8. Incubation was carried out in a shaking water bath (Grant OLS, Cambridge, UK), and the sample was allowed to cool, mixed thoroughly and then centrifuged at 2300×*g* for 20 min. The resulting mixture separated into four layers; a solid residue at the bottom, an aqueous skim layer, a creamy emulsion layer and a clear oil layer at the top. At least 2–3 tubes were used per bottle and so top layers of the tubes were decanted into a different tube and centrifuged for 20 min at 4000 rpm to separate the emulsion/free oil layer. The emulsion/free oil layer was refrigerated overnight and the oil was collected from the top of the emulsion layer. A study conducted in our laboratory showed that a mixture of α-amylase, Alcalase and Celluclast gave highest yields compared to individual preparations.

High pressure processing was used as a pre-treatment before AEE (HPP-AEE). The samples were vacuum sealed in polyethylene bags and placed in a pressure vessel (Stanstead Fluid Power, Ltd) and subjected to a pressure of 300 MPa. Temperature and time were maintained at 25 °C and 20 min, respectively. A mixture of water and 1,2-propanediol (70:30, v/v) served as the pressure transmitting fluid. Studies in our laboratory showed that a pressure of 300 MPa exerted on tiger nut samples before AEE increased oil yields.

Extracted oils were stored in amber bottles and maintained at 4 °C till analysis.

## Chemicals

Supelco 37 Component fatty acid methyl ester (FAME) Mix, α-tocopherol standard, gallic acid, phenolic acid standards (vanillic acid, *trans*-ferulic acid, vanillin, and *trans*-cinnamic acid), glucose, 1-kestose (DP3) and nystose (DP4) standards were purchased from Sigma-Aldrich (Dorset, UK). All chemicals were of analytical grade.

## Oil Analysis

### HPLC Phenolic Profile

Phenolic extracts to be used for HPLC analysis were obtained using the liquid–liquid extraction procedure outlined by Pirisi, Cabras, Cao, Migliorini and Muggelli [7]. Oils were weighed into tubes with 1 ml hexane and 2 ml methanol (6:4, v/v) and vortexed for 2 min. The mixture was centrifuged at 2300×*g* and the methanol layer was separated. This was repeated twice and the extract was washed with 2 ml hexane. Methanolic extracts were evaporated to dryness at 35 °C and re-dissolved in 1 ml methanol prior to injection.

Analysis was carried out with a HPLC–DAD system (Agilent 1200, Manchester, UK) using a Nova-Pak C-18 reverse phase column (4 µm, 25 cm × 4.6 mm i.d.) (Waters Limited, Hertfordshire, UK). Mobile phases were 0.001 M H_2_SO_4_ (A) and acetonitrile (B) at a flow rate of 1 ml/min. The detector was set at 225 nm and the sample loop was 20 μl volume. The gradient program was as follows: *t* = 0 min, *A* = 85 %, *B* = 15 %; *t* = 35 min, *A* = 34 %, *B* = 66 %; *t* = 35.1–40 min, *A* = 85 %, *B* = 15 %. Identification of phenolic compounds was done by comparison with peak times and spectra of standards. An external calibration curve was constructed with five standard solutions and used for quantification.

### Total Polyphenol Content

The extraction and quantification of phenols was carried out using the procedure described by Baiano *et al*. [8]. 2 milliliters of methanol/water (70:30, v/v) and 2 ml of hexane were added to 5 g of tiger nut oil and vortexed for 10 min. The organic phase and the aqueous phase were separated by centrifugation (6000 rpm, 4 °C, 10 min). The aqueous phase containing the phenolics was collected and centrifugation was repeated (13,000 rpm, room temperature, 4 min). Finally, the aqueous phase was collected with a pipette for analysis. The phenolic extract (100 µl) was mixed with Folin-Ciocalteu reagent (100 μl) and after 4 min, 800 μl of 5 % Na_2_CO_3_ was added. The mixture was incubated at 40 °C for 20 min, after which the absorbance was determined at 750 nm. A five point calibration curve using gallic acid in methanol/water (7:3, v/v) was constructed and TPC was expressed as gallic acid equivalents (GAE) per kg oil.

### Tocopherol Content

The method described in Ezeh *et al*. [4] was used. β-Tocopherol is expressed as an α-tocopherol equivalent. To extract tocopherols, 0.4 g oil was homogenised with 4 ml of 75:25 isopropanol:chloroform solution. Analysis was performed with a HPLC–UV system (Agilent 1200, Manchester, UK) using a Nucleosil C-18-100 reverse phase column (25 cm × 4.6 mm i.d.) with a particle size of 5 µm (Macherey-Nagel, Duren, Germany). The injection volume was 10 µl and the mobile phase was 67:27:6 (v/v/v) methanol:tetrahydrofuran:water. Detection was set at 292 nm and flow rate was maintained at 0.8 ml/min. Dilute concentrations of α-tocopherol standard were prepared by dissolving in methanol. Tocopherol was identified by comparing the retention times with those of the standards and comparing the absorption spectra obtained by the DAD. An external five point calibration was used for quantification.

### Fatty Acid Profile

Oils were analysed for fatty acid composition by Gas Chromatography (Agilent HP 6890 fitted with a flame ionisation detector) and the procedure in Ezeh *et al*. [4] was followed. Fatty acid methyl esters were prepared by saponification as described in the International Union of Pure and Applied Chemistry method 2.301 [9]. The esters were analysed using a fused silica capillary column Varian CP-Sil 88 (50 m × 0.25 mm × 0.20 µm). The injector temperature was 250 °C; detection temperature was 260 °C and oven temperature was initially 100 °C, held for 3 min and ramped to 240 °C at 4 °C per min. The carrier gas was hydrogen at a flow rate of 0.8 ml/min. The fatty acids were identified by comparing retention times with those of standards.

### Free Fatty Acid and Peroxide Values

Acid values (AV) were measured using the method described in Ezeh, Gordon and Niranjan [4] and converted into the percentage of free fatty acids (FFA) using the formula % FFA = AV × 1.99.

For peroxide values (PV), the International Dairy Federation (IDF) method for determination of peroxide value was adopted [10]. Oils (0.3 g) were weighed in test tubes and 9.8 ml of chloroform/methanol (7:3, v/v) was added. The mixture was vortexed for 2–4 s, followed by addition of ammonium thiocyanate (50 µl) solution. The sample was vortexed again for 2–4 s and 50 μl of iron (II) solution was added, vortexed and left to incubate for 5 min at room temperature. After incubation, the absorbance was taken at 500 nm against a blank. The blank was a mixture containing all reagents without oil sample. Analysis was conducted under limited lighting. A calibration curve of Fe^3+^ concentration against absorbance was constructed using five standard solutions of iron (III) chloride (5–40 µg Fe^3+^).

1 $${\text{Peroxide value was calculated using the formula}}\;{\text{PV}} = \frac{{\left( {A_{\text{s}} - A_{\text{b}} } \right) \times m}}{{55.84 \times \,m_{ 0} \times 2}}$$

where *A*_s_ = absorbance of sample; *A*_b_ = absorbance of blank; *m* = slope of calibration curve; *m*_0_ = mass of sample in grams; 55.84 = atomic weight of iron.

## Residual Meal Analysis

### Chemical Analysis

A modified version of the Kjeldahl method AOAC 955.04 [11] was used to measure protein content. Ground tiger nut samples were weighed out into a digestion tube with 8 g of catalyst added. Concentrated sulphuric acid (25 ml) was added and heated for approximately 45 min till the solution was clear. It was removed and left to cool before distillation. Distillation was carried out using 50 ml water and 125 ml NaOH in a distillation unit. A conical flask containing 50 ml 2 % boric acid and few drops of screened methyl red was placed in the distillation unit to receive the condensate. The collected mixture in the conical flask was titrated against 0.01 N sulphuric acid. Percentage nitrogen was calculated using the following formula.

$$\% {\text{N}} = {\text{N}}\,{\text{H}}{}_{2}{\text{SO}}_{4} \, \times \,\left[ {\frac{{{\text{ml}}\,{\text{titrant}}\,{\text{volume}}\, ( {\text{sample)}} - {\text{ml}}\,{\text{titrant}}\,{\text{volume}}\, ( {\text{sample)}}}}{{{\text{sample}}\,{\text{weight}}\, ( {\text{g)}}}}} \right] \times 14\,{\text{g}}\frac{\text{N}}{\text{mol}} \times 100$$

where 14 is the molecular weight of nitrogen. The percentage of nitrogen was converted to total protein using 6.25 as a conversion factor for tubers. Soxhlet extraction was used for sample preparation for oil measurements. Ash content was determined using AOAC method 942.05 [11], moisture using a Sartorius moisture analyser and total carbohydrate was calculated by difference; 100 % −  % (crude protein + ash + crude fat + moisture).

### Extraction of Sugars

Residual meals from oil extraction were defatted and soluble sugars were extracted by a modified method based on Teixeira *et al*. [12]. A sample (50 mg) was placed in a centrifuge tube and 2.5 ml of 80 % ethanol was added. The mixture was vortexed for 5 s and heated on a water bath at 95 °C for 20 min. Following this, it was centrifuged at 4000 rpm for 20 min and the extraction was repeated three times. The ethanolic extracts were combined and evaporated under pressure at 42 °C till dryness. Extracted sugars were re-dissolved in 2.5 ml deionised water, filtered (0.45 µm) and kept at −20 °C till analysis.

#### Quantification of Sugars

HPLC analysis was performed with an Agilent 1100 system (Cheshire, UK) coupled to a refractive index detector. The mobile phase was HPLC grade water with a flow rate of 0.25 ml/min. The stationary phase was an Aminex HPX-42A column, 7.8 × 300 mm (Bio-Rad) attached to an anion-exchange guard column. The column was found to hydrolyse high DP sugars at high temperatures and as a result, the temperature was maintained at 25 °C. Peaks were identified by comparison with the retention times of external standards. To quantify the sugars, a five point calibration curve was constructed.

To further identify the sugars present, the samples were analysed using a Dionex ion chromatography system (Sunnyvale, CA) consisting of ED50A pulsed amperometric detector (PAD) operating in the integrated amperometry mode, AS50 autosampler and GS50 gradient pump. The system was also equipped with a CarboPac PA1 (4 × 250 mm) analytical column and a CarboPac PA1 (4 × 50 mm) guard column (Dionex Corp., Sunnyvale, CA). The mobile phase was HPLC grade water (A), 1000 mM NaAc (B) and 500 mM NaOH (C). The following profile was used; 0–30 min, 80 % A, 20 % B; 30–35 min, 63 % A, 17 % B, 20 % C; 35–40 min, 60 % A, 20 % B, 20 % C; 40–41 min, 22.5 B, 20 % C; 41–46 min, 80 % B, 20 % C; 47–65 min, 80 % A, 20 % C. The injection volume was 20 μl and the flow rate was maintained at 1.0 ml/min at room temperature. Peaks were identified using available sugar standards. Data analysis was performed using a Chromeleon V6.8 (Dionex).

### Statistics

All experiments were performed in triplicate and mean values are presented with their standard deviations. Statistical analysis was done using Microsoft Excel 2010 and SPSS Version 20 Statistical software (SPSS Inc, Chicago, USA). Significance was defined at *p* < 0.05.

## Results and Discussion

### Phenolic Compound Profile

**Fig. 1:**
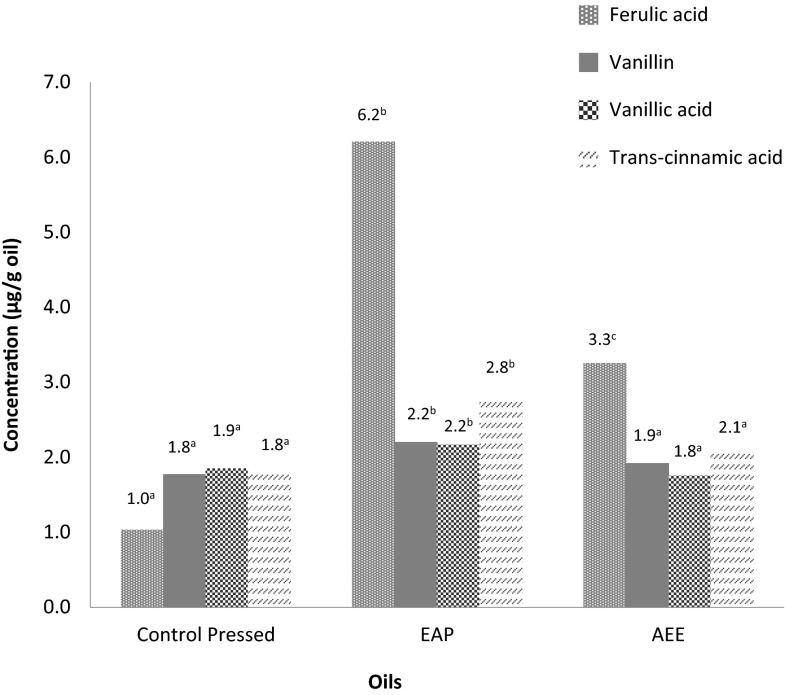
Phenolic profile of tiger nut oils extracted using different methods; *EAP* enzyme aided pressed (enzyme concentration of 1 %, incubation time of 6 h, S/L ratio 1:1.7, pressing time of 30 min, maximum pressure of 38 MPa); *AEE* aqueous enzymatic extracted (enzyme concentration of 1 % (w/w), incubation time of 4 h, S/L ratio 1:4) different superscript letters across oils for the same phenolic compound indicate significant differences

Enzyme aided pressed (EAP) and aqueous enzymatic extracted (AEE) oils were analysed for their phenolic contents using HPLC–DAD. Four simple phenolic compounds were identified; *trans*-ferulic acid, vanillic acid, vanillin and *trans*-cinnamic acids. There were some peaks that could not be identified due to the limitation of DAD detectors as they require known standards. Enzymatic pre-treatment increased the concentration of all phenolic compounds, most especially for *trans*-ferulic acid which had the highest concentration of the phenolic compounds at 6.2 μg/g (Fig. 1). The hemicellulase mixture, Viscozyme was most likely responsible for the increase in *trans*-ferulic acid. Ferulic acid exists as an esterified component of tiger nut cell wall where it cross-links hemicellulose polymers such as arabinoxylans [13]. Hydrolysis of these ester linkages would result in the release of the bound ferulic compound.

In AEE extracted oil, the quantities of phenolic compounds were slightly lower except *trans*-ferulic acid which was about half of that present in EAP oils. For AEE, Celluclast instead of Viscozyme was used, which may explain the lower ferulic acid content as cellulose was the major part of the cell wall that was acted upon. Vanillin has been identified previously in roasted tiger nut oil [14] as one of the key compounds responsible for the sweet vanilla aroma of the oil. This property of tiger nut oil can be taken advantage of, both for cosmetic and edible food applications.

### Total Polyphenolic Content

**Fig. 2:**
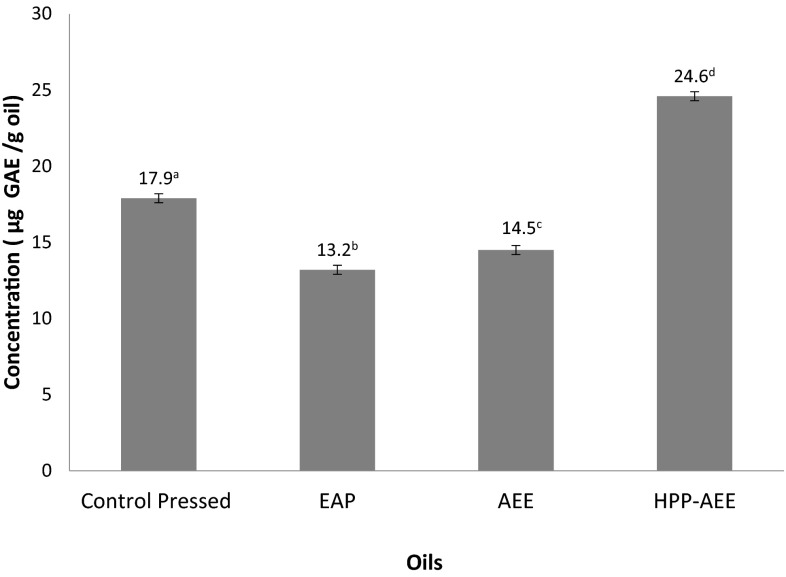
Total polyphenol content of tiger nut oils extracted using different methods; *EAP* enzyme aided pressed oil (enzyme concentration of 1 %, incubation time of 6 h, S/L ratio 1:1.7, pressing time of 30 min, maximum pressure of 38 MPa); *AEE* aqueous enzymatic extracted oil (enzyme concentration of 1 % (w/w), incubation time of 4 h, S/L ratio 1:4); *HPP*-*AEE* high pressure processing-aqueous enzymatic extracted oil (HPP for 20 min at 25 °C); different superscript letters across oils indicate significant differences

Amongst the oils assessed, HPP-AEE oil contained the highest concentration of polyphenolic compounds as shown in Fig. 2. The impact of high pressure appears to have greatly enhanced the release of phenolic compounds into the aqueous enzymatic extracted oil. On the other hand, the use of enzyme as a pre-treatment decreased the total polyphenolic content (TPC) in pressed oil. The vacuum drying step may have contributed to the loss of polyphenolic compounds either via action of oxidative enzymes such as polyphenol oxidase. Suvarnakuta *et al*. [15] observed that xanthones, a group of polyphenols in mangosteen decreased significantly when vacuum dried at 60 °C while hot air drying and vacuum drying at higher temperatures retained more of the polyphenols. The lower temperature of 50 °C used during vacuum drying of tiger nut before pressing may be insufficient to inactivate these enzymes, making it more likely for oxidative degradation to occur thus resulting in lower TPC in EAP oil. TPC has been linked to the antioxidant capacity of oils as polyphenols act as free radical scavengers [8]. HPP-AEE oil may thus have a higher antioxidant capacity than other oils extracted.

### Tocopherol Content

**Fig. 3:**
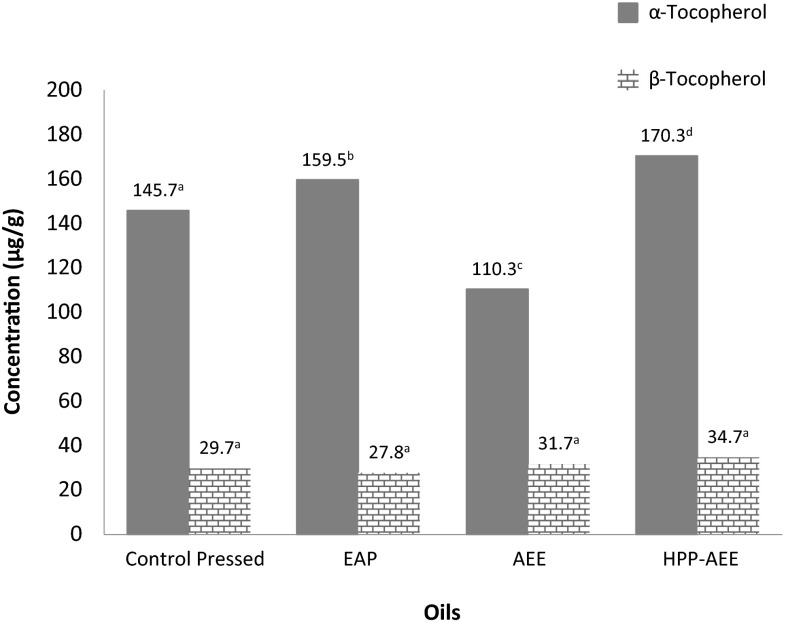
Tocopherol content of tiger nut oils extracted using different methods; *EAP* enzyme aided pressed oil (enzyme concentration of 1 %, incubation time of 6 h, S/L ratio 1:1.7, pressing time of 30 min, maximum pressure of 38 MPa); *AEE* aqueous enzymatic extracted oil (enzyme concentration of 1 % (w/w), incubation time of 4 h, S/L ratio 1:4); *HPP*-*AEE* high pressure processing-Aqueous enzymatic extracted oi; (HPP for 20 min at 25 °C); different superscript letters across oils for each tocopherol indicate significant differences

The α-tocopherol content in EAP oil was higher than that of control pressed oil. The use of enzymes in pressing likely increased the amount of tocopherol released due to hydrolysis of cellular structures. As for TPC, the highest quantity was present in HPP-AEE oil as can be observed in Fig. 3. From a previous study in our laboratory, HPP as a pre-treatment increased oil yields by increasing starch hydrolysis which was found to affect oil yields positively. This allowed easier access of oil bodies out of tiger nut cells and consequentially, may have contributed to the increase in tocopherol content. β-tocopherol content was similar across both pressed samples and not very different between AEE samples. In general, the content of β-tocopherol was much less compared to α-tocopherol for all samples. Of the two identified tocopherols, α-tocopherol is the only one that contributes to daily human Vitamin E requirements, as it is preferentially absorbed and incorporated into membranes by the body.

The quantities of α-tocopherol found in tiger nut oil (except AEE oil) surpassed those measured in a different study involving tiger nut oil with only 86.7 mg/kg [3]. Additionally, it is higher than α-tocopherol in canola (117–125 mg/kg), soybean (64–75 mg/kg) and corn oil (122–129 mg/kg), oils commonly found in supermarkets [1], and hence used by consumers, restaurants and in the frying industry. Consequently, tiger nut oil can be used as a source of Vitamin E and even expanded to be employed in skin care for a similar purpose (source of Vitamin E).

### Fatty Acid Profile

The fatty acid (FA) profiles for the extracted oils are shown in Table 3. There was minimal variability between all samples extracted and pre-treatments did not seem to affect the concentration of the acids. Oleic acid was the most abundant FA and the main monounsaturated FA present. As was mentioned before, when describing the FA content of pressed oil only, minor quantities of myristic, linolenic acid and arachidic acids were found in all oils making them comparable to previously reported values for tiger nut oil [16]. All samples also have the same benefits already described for pressed tiger nut oil with regards to their high concentrations of monounsaturated FAs.

**Table 3:** Fatty acid profile of tiger nut oils

Fatty acid	Control pressed (%)	EAP (%)	AEE (%)	HPP-AEE (%)
C14:0	0.1 ± 0.00^a^	0.1 ± 0.00^a^	0.1 ± 0.00^a^	0.1 ± 0.00^a^
C16:0	13.5 ± 0.00^a^	14.5 ± 0.08^b^	13.9 ± 0.06^a^	13.7 ± 0.00^a^
C16:1	0.3 ± 0.00^a^	0.3 ± 0.00^a^	0.0 ± 0.00^b^	0.3 ± 0.00^a^
C18:0	6.3 ± 0.03^a^	6.6 ± 0.08^b^	6.4 ± 0.04^b^	6.2 ± 0.03^a^
C18:1	67.4 ± 0.07^a^	66.0 ± 0.04^a^	66.1 ± 0.07^a^	66.0 ± 0.41^a^
C18:2	10.7 ± 0.05^a^	11.0 ± 0.07^a^	11.6 ± 0.05^b^	12.0 ± 0.05^b^
C18:3n6	0.1 ± 0.00^a^	0.1 ± 0.01^a^	0.1 ± 0.00^a^	0.1 ± 0.00^a^
C20:0	0.7 ± 0.01^a^	0.6 ± 0.01^a^	0.7 ± 0.01^a^	0.7 ± 0.01^a^
C20:1	0.1 ± 0.00^a^	0.1 ± 0.03^a^	0.1 ± 0.00^a^	0.1 ± 0.00^a^
C24:0	0.2 ± 0.01^a^	0.2 ± 0.01^a^	0.3 ± 0.01^a^	0.3 ± 0.01^a^
Unknown	0.4 ± 0.02^a^	0.4 ± 0.03^a^	0.4 ± 0.02^a^	0.4 ± 0.02^a^

Values are expressed as means and standard deviations, *n* = 3, different superscript letters across each row indicate significant difference

### Free Fatty Acid and Peroxide Values

The percentage of free fatty acids is one good indicator of the quality of the oil. A high value reflects that the quality of the oil is reduced [3]. All samples shown in Table 4 had a percentage value lower than 2.0 % thus meeting the criteria set for virgin olive oils by the International Olive Oil Council (IOC). However, they did exceed the % FFA standard for extra virgin olive oil of <0.8 % with the exception of AEE oil [17]. Storage conditions and time between harvests of tiger nuts, drying time and their purchase are unknown and hydrolytic reactions leading to free fatty acid production may have already begun in the nuts, since there were no significant difference between oils subjected to different treatments.

**Table 4:** Free fatty acid and peroxide values of tiger nut oils

	Control pressed	EAP	AEE	HPP-AEE
% FFA	1.8 ± 0.19^a^	1.7 ± 0.01^a^	1.63 ± 0.09^a^	1.82 ± 0.28^a^
PV (mequiv O_2_/kg oil)	1.2 ± 0.02^a^	1.68 ± 0.01^b^	0.13 ± 0.13^c^	1.38 ± 0.01^a^

Values are expressed as means and standard deviations, *n* = 3, different superscript letters across each row indicate significant difference

On the other hand, all the peroxide values (PV) fall well below PV standards set by IOC for olive oil of <20 mEqO_2_ per kg oil. The low values also suggest low progression of any oxidative rancidity in the oils. Enzyme pre-treatment prior to pressing increased PV slightly, while HPP had a larger effect on AEE oil.

## Residual Meals

### Chemical Analysis

Table 5 shows the composition of tiger nut meals after oil extraction. AEE had the highest oil and lowest protein content. The high oil content in the meal is a consequence of the lower oil yield achieved with AEE compared to EAP, indicating that it is not an efficient method of extracting tiger nut oil. The lower protein content in the might be due to a larger degree of protein hydrolysis. With the higher solid–liquid ratio used in AEE compared to EAP, more water is available for protein hydrolysis to take place effectively. Similar reasoning may also explain the lower carbohydrate content including the fact that a different carbohydrase was used.

**Table 5:** Composition of tiger nut meals (dry basis) and oil yields from process

Sample	Control pressed	EAP	AEE
Crude fat	8.0 ± 0.80^a^	5.1 ± 0.02^a^	20.3 ± 1.29^a^
Crude protein	4.1 ± 0.21^a^	4.6 ± 0.19^a^	2.4 ± 0.04^b^
Ash	2.6 ± 0.01^a^	3.2 ± 0.04^a^	1.9 ± 0.41^b^
Total carbohydrate	74.7 ± 0.23^a^	76.4 ± 0.33^a^	65.8 ± 0.15^b^
Oil yield (%)	61.3 ± 0.02^a^	89.3 ± 1.04^b^	61.3 ± 0.04^a^
Solids (%)	91.2 ± 0.80^a^	94.6 ± 0.02^b^	76.6 ± 0.50^c^

Values are expressed as means and standard deviations, *n* = 3, different superscript letters across each row indicate significant differences

EAP with the highest protein and lowest oil content reflects the highest oil yield that was achieved with this method of oil extraction. Notwithstanding the method used, the protein contents in all samples were lower than those in major oil seed meals such as soybean, canola, and sunflower with typical protein content of 47.5, 35.6, and 42.2 %, respectively [18]. One should bear in mind that tiger nut is a tuber with a low protein content of 3.2 % so its meals would also have low protein levels. As a consequence, tiger nut meals would be deemed unsuitable for animal or fish feeding.

### Sugars in Residual Meals

To assess a prospective use for the by-products of the oil extraction process, soluble sugars in the residual meals were determined. Meals remaining from EAP and AEE were the only samples found to contain peaks that appeared to be sugars with low degree of polymerisation (DP). Both DP3 and DP4 sugars were present in EAP meal and only DP3 sugars in AEE meal. All samples except CP meal contained sucrose, fructose and glucose as shown in Table 6. Using HPAEC-PAD, the sugars coincided with the peak times of the standards nystose and 1-kestose. These are short chain length fructans comprising of linear chains of α-d-glucopyranosyl-[β-d-fructofuranosyl] *n*-1-β-fructofuranoside (GFn) [19]. Higher quantities of sugars with similar DP as 1-kestose were quantified in the EAP meal using the BioRad column as shown by the areas of the peaks from the HPAEC-PAD profiles. Traces of possible higher DP sugars were observed in the HPAEC-PAD profile of EAP meals that were not completely resolved using the BioRad column and would thus require standards for verification.

**Table 6:** Sugars in residual meals of tiger nuts after oil extraction

Sample	Mean ± SD (mg/g, dry basis)				
	Fructose	Glucose	Sucrose	DP3	DP4
Control	<dl	<dl	246.5 ± 1.02^a^	<dl	<dl
EAP	20.4 ± 0.48^a^	63.1 ± 0.85^a^	80.3 ± 0.45^b^	73.2 ± 1.05^a^	9.3 ± 0.60^a^
AEE	30.8 ± 1.12^b^	8.4 ± 1.08^b^	50.0 ± 0.76^c^	11.1 ± 0.65^b^	0.0^b^

Values are expressed as means and standard deviations, *n* = 3, different superscript letters in each column indicate significant differences

*dl* detection limit

The results in Table 6 suggest that the actions of enzymes raised the yield of soluble higher DP sugars in the meals. The difference between EAP and AEE samples was the type of carbohydrase used. EAP employed Viscozyme, a mixture of carbohydrases including xylanase, arabanase and hemicellulase while AEE was done with Celluclast, predominantly a cellulase. Having an array of different enzymes in Viscozyme allowed for hydrolysis of different components of tiger nut cell walls, and helped to increase the release of short chain sugars.

Although Pollard [20] detected fructose oligosaccharides (FOS) in plants belonging to the Cypereae tribe, the same tribe that tiger nuts belong to, a tribe being a taxonomy rank between family and genus, it should be noted that short chain sugars may be products of polysaccharides degradation. During the enzymatic treatment, starch for example was hydrolysed and products such as the oligosaccharide maltotriose with a DP of three could be released. Arabinoxylan oligosaccharides may be another possible explanation for these sugars. In general, plant oligosaccharides are considered to be beneficial to human health including the immune system. They may stimulate the growth of bifidobacteria and lactobacilli as well as the production of short chain fatty acids by the gut microbiota [21]. These fatty acids have been shown to exert favourable effects on mammalian energy metabolism [21]. Nonetheless, the oligosaccharides in tiger nut meal would require further qualitative assessment to confirm their identity. An evaluation of their functional characteristics and effects on human health and well-being would also be required in order to safely recommend them for use. Tiger nut residual meals may potentially serve as a source of possible valuable prebiotics.

## Conclusion

Oils extracted using EAP showed increased content of key bioactive compounds such as tocopherol and some phenolic acids. HPP-AEE oil also had higher tocopherol and TPC content compared to AEE oil. The quality parameters (% FFA and PV) of the oils indicated that they were all under the IOC recommended values for virgin olive oil, thus are good quality and quite stable oils. Their fatty acid profiles remained unchanged by processing conditions used.

Residual meals obtained from the process showed that all samples contained low levels of protein. In particular, high oil content of AEE meal showed the low efficiency of the technique in extracting tiger nut oil. Sugar analysis of EAP and AEE samples showed that they contained 82.5 and 11.1 mg/g sugars with DP3 and DP4 chain lengths, respectively. Further analysis would be required to confirm the identity of these sugars as well as their potential quality including their effect on human health.
